# The Silent Epidemic of Exclusive University Licensing Policies on Compounds for Neglected Diseases and Beyond

**DOI:** 10.1371/journal.pntd.0000570

**Published:** 2010-03-30

**Authors:** Connie E. Chen, C. Taylor Gilliland, Jay Purcell, Sandeep P. Kishore

**Affiliations:** 1 School of Medicine, University of California, San Francisco, California, United States of America; 2 Universities Allied for Essential Medicines (UAEM), Berkeley, California, United States of America; 3 Biomedical Sciences Graduate Program, University of California, San Diego, La Jolla, California, United States of America; 4 Berkeley Law School, University of California, Berkeley, California, United States of America; 5 Tri-Institutional MD–PhD Program, Weill Cornell/Rockefeller/Sloan-Kettering Institute, New York, New York, United States of America; New York Blood Center, United States of America

In 2007, the University of British Columbia (UBC) unveiled a new licensing strategy to promote developing world access to its technologies [1]. UBC's “Global Access License Principles” aim to ensure that the university's biotechnology and environmental licensees develop and market UBC-derived technologies for global benefit. Shortly thereafter, UBC licensed rights to commercialize a low-cost, oral formulation of Amphotericin B developed by Dr. Kishor M. Wasan. It was the first technology licensed under the new policy. In addition to its improved ease of administration and reduced toxicity when used as an antifungal agent, Dr. Wasan's oral Amphotericin B is also a novel agent against leishmaniasis. In return for rights to market the drug for the treatment of blood-borne fungal infections in the developed world, iCo Therapeutics agreed to produce and sell at-cost versions of the drug for treatment of leishmaniasis in developing countries.

UBC's innovative licensing strategy for novel leishmaniasis treatment illustrates the powerful role university licensing agreements can play in expanding global medicine access. However, most other universities have yet to fully replicate the University's strategy in establishing a global access pathway to bring their life-saving technologies to patients. We present here a case study describing current discussions at the University of California (UC) system on the adoption of global access licensing (GAL) principles. Such reform could promote developing-world access to the breadth of future UC-developed technologies, and motivate change at peer institutions. Globally, the recent expansion of Bayh-Dole–like legislation—a United States law that enables universities to exclusively license technologies derived from federally funded research—highlights the additional importance of GAL principles for institutions in the global South, notably in India and South Africa. Bayh-Dole–style laws currently under consideration in those countries contain weak provisions for safeguarding public access to publicly supported medicines [2],[3]. The continued expansion of such laws to nations heavily engaged in neglected disease research could have profound impacts on access to the products that result from their work [4].

## Global Access Licensing

Humanitarian or global access licenses have achieved striking effects. One widely advocated iteration involves licensees voluntarily allowing generic production of the final product for exclusive distribution in low- and middle-income (LMI) countries. This approach realizes economies of scale and market competition by locating the most efficient manufacturer and distributor. In 2001, Yale University and Bristol-Myers Squibb agreed to that situation for its widely used HIV drug stavudine (d4T, Zerit), triggering a 96% price reduction of the drug in South Africa. Simply by permitting the manufacture and sale of generic stavudine in South Africa, market forces drove down the price of the small molecule [5]. Importantly, Yale's action came at no cost to the university in terms of licensing revenues. As much was reiterated by Yale's Dean of Public Health Michael Merson after Yale disclaimed stavudine royalties: “[t]his change was made at Yale without any negative consequences for the University—financial or otherwise” [6]. This case example demonstrates the viability of utilizing university technology transfer to accelerate access to medicines.

Currently, other universities have adopted similar licensing approaches to varying effects including: the University of British Columbia (as previously described), Emory University, University of Edinburgh, University of Oxford, University of Washington, the University College of London, and Boston University [7],[8],[9],[10],[11],[12]. Example access strategies enumerated in Emory's Global Access Principles include structuring diligence obligations to facilitate developing-world access to low-cost products and encouraging licensees to sublicense or forego patent protection in developing countries.

Ironically, in failing to modify their licensing practices, most major research universities have fallen behind their industry licensing partners. Many pharmaceutical and biotechnology companies acknowledge sublicensing to generic producers as a socially responsible and financially viable method to supply medicines to low-margin developing world markets. In its Global Access Program, Gilead Sciences has partnered with 13 Indian pharmaceutical companies to enable the production and distribution of generic versions of its HIV medication tenofovir (Viread) for 95 developing countries [13]. Eli Lilly granted a sublicense for the manufacture of generic versions of two antibiotics effective against multidrug-resistant tuberculosis (MDR-TB) by companies in South Africa, China, India, and Russia [14]. GlaxoSmithKline (GSK) granted licenses to a number of generic companies for the manufacture and sale of generic antiretrovirals (ARVs) across sub-Saharan Africa, including most recently abacavir (Ziagen), a therapy derived from compounds first synthesized at the University of Minnesota [15]. In its corporate responsibility report published in March 2009, GSK committed to placing over 800 granted and pending patents in a patent pool to help others to develop potential medicines for neglected diseases [16]. Alnylam Pharmaceuticals followed suit in July 2009, adding 1,500 patents to the GSK pool [17].

But because many other pharmaceutical firms persist in using traditional licensing techniques, universities like the UC are uniquely positioned to leverage their ownership rights and manage their intellectual property so that patent, data exclusivity, and other legal barriers do not unnecessarily prevent generic manufacturers from supplying much-needed drugs to impoverished countries at the lowest possible cost.

## Universities Allied for Essential Medicines

To maintain this pressure, the international student-run organization, Universities Allied for Essential Medicines (UAEM; http://www.uaem.org), promotes access to drugs that flow from publicly financed research. UAEM recognizes developed-world patents as important drivers of innovation, provided that universities engage in responsible intellectual property management that consciously furthers global-access objectives. In 2008, UAEM members successfully included this clause in the Democratic National platform: “We also support the adoption of humanitarian licensing policies that ensure medications developed with the U.S. taxpayer dollars are available off patent in developing countries” [18]. For his 2008 Yale Law School Alumni Address, Former President Bill Clinton further stated that “universities ought to take the lead in…[using] their discoveries under conditions that will guarantee affordable medicines that save millions of lives.”

In December 2008, in consultation with academic experts and technology transfer officers, UAEM released a Framework to inform the development of institutional access policies (see Box 1). Though concern persists among university administrators that pharmaceutical sales (and university royalties) might be negatively impacted by a GAL, economic realities and market segmentation suggest that it would not diminish revenue streams. A GAL would only guide licensing in LMI countries in which major pharmaceutical companies revenues and profits are scarce. According to industry reports, consumers in the US, Canada, the European Union, and Japan contribute to 93.2% of all pharmaceutical revenues [19].

**Box 1. The Global Access License (GAL) Framework**

Universities should implement Global Access Policies that adhere to the following five principles:

1. Access to medicines and health-related technologies for all is the primary purpose of technology transfer of health-related innovations.
2. Technology transfer should protect access to the final end product needed by patients (e.g., formulated pills or vaccines).
3. Generic provision is the best way to ensure access to medicines in resource-limited countries. Legal barriers to generic production of these products for use in resource-limited countries should therefore be removed.
4. Proactive licensing provisions are essential to ensure that follow-on patents and data exclusivity cannot be used to block generic production.
5. University licensing should be systematic in its approach, sufficiently transparent to verify its effectiveness, and based on explicit metrics that measure the success of technology transfer by its impact on access and continued innovation.

While countries where a GAL would facilitate access have few consumers, they contain the world's vast majority of patients. Ninety percent of the world's poor live in Africa, China, and India, but together constitute just over 1% of the global branded pharmaceutical market [16]. Individual patients, as well as the governments and nongovernmental organizations that often purchase medicines on their behalf, here are simply too resource-limited to afford brand-name treatment and instead rely on low-cost generic medicines. Mozambique, for instance, is home to at least 1,500,000 HIV-positive persons, yet per capita gross national income is $320 [20],[21]. Despite poverty reduction efforts, Mozambicans are not likely to become consumers of full-priced drugs, vaccines, or diagnostics in the near or medium term. Indeed it is doubtful whether poverty can be overcome without access to improved health care [22]. The only revenue that could come from treating these persons with pharmaceuticals will originate in funding agencies—the Global Fund, UNITAID, PEPFAR, GAVI, UNICEF—whose impact depends largely upon their ability to negotiate at- or low-cost prices.

## Spotlight on the University of California

The adoption of GAL licensing terms may be nowhere more relevant than at UC, a public institution reaping nearly 10% of the US National Institutes of Health (NIH) external research budget, making it the largest recipient of NIH funding in the U.S. Moreover, industry reports now show that, of all institutions of higher education, the UC system averaged the highest level of licensing income annually from its research discoveries in biotechnology from 1997 to 2003 [23]. This follows from the fact that research laboratories of the university's ten campuses produce more biotech patents than any other similar organization in America, including the US government, Genentech, and the University of Texas [24]. In fact, from 2002 to 2006, UC was second worldwide only to the Japan Science and Technology Agency in number of biotechnology patents. Key UC discoveries include the hepatitis B vaccine; Fuzeon, a salvage HIV therapy; and a low-cost synthetic method of producing artemisinin for malaria.

To date, substantive global-access licensing practices similar to those of Emory or UBC have yet to be drafted or implemented across the UC system. One objection relates to infeasibility of therapy scale-ups, and fear of driving drug resistance through poor adherence. Another is economic, and dwells on risks of pharmaceutical arbitrage and reimportation of generic products to developed markets. To the first point, though nonadherence and the development of drug resistance are associated, a review of over 55 studies around the world indicates that patients in the developing world achieve rates of adherence (for both TB and HIV therapy) superior to patients in the most developed of settings [25],[26]. Concerns over poor-quality drugs, compliance with incorrect regimens, and transmission of drug-resistant disease strains are of concern in decisions to increase drug distribution, but are currently being addressed and monitored by leading public health officials at the World Health Organization (see http://www.who.int/medicines/areas/rational_use/en/ for more). To the second point, while pharmaceutical arbitrage certainly looms as a threat, it remains a mostly theoretical and rarely empirically observed phenomenon [27]. Conventions adopted by the World Trade Organization allow for unique pill shape, color, and packaging to distinguish generic formulations from brand-name pharmaceuticals [28],[29]. Moreover, international mechanisms of legal recourse exist for those individuals and firms who break the law. Finally, Gilead's own practices demonstrate the rationality of generic sublicensing for some products, as existing manufacturing capacity likely cannot meet the vast, low-margin demand accompanying scale-up of essential medicines in the developing world.

At the same time, negotiations and lobbying with UC continue; in June 2009, UC established a subcommittee to consider global-access principles. To date, however, no timeline or deliverables are defined or slated. Given its immense research output as well as its history of promoting access to their discoveries with industry, we believe that the UC system can lead its peer institutions in adopting GAL principles (see Box 2).

**Box 2. UC Success Stories: Global Access to Hepatitis B vaccine and Synthetic Artemisinin**

Three Bay Area scientists (William Rutter, Edward Penhoet, and Pablo Valenzuela) founded Chiron, and along with researchers at UCSF developed and licensed the key ingredient (hepatitis B surface antigen) used in Recombivax HB, Merck's hepatitis B vaccine. Through a progressive technology transfer agreement and one-time fee of US$7 million to the Chinese government, Merck trained Chinese scientists and engineers in vaccine production in the US. Next, Merck chemical engineers accompanied the Chinese teams to help scale up two state-of-the art recombinant DNA vaccine plants, in Beijing and Shenzhen, capable of producing 20 million doses a year (to immunize all newborns). This deal was precipitated by Merck's realization that the Chinese could not afford the vaccine at even a reduced price with no effect on developed world market vaccine revenues [35]. Years later, UC Berkeley negotiated royalty-free license for a novel, low-cost artemisinin production process signed with the Institute for One World Health, drawing wide praise and $42 million in support from the Gates Foundation [36].

## Global Access Licensing at All Universities

Ultimately, we envision universal adoption of Global Access Licensing via the GAL Framework by all research institutions, so that these concepts inform all relevant licenses. UAEM is sensitive to the fact that licenses are complex and each will be unique. Its Framework does not prescribe specific language; rather, its five principles can guide university-specific policy. We believe that these changes are so fundamental to global health that their implementation cannot wait.

There are signs of progress. On November 9th, 2009 six universities (Harvard, Yale, Boston University, Oregon Health Sciences University, the University of Pennsylvania and Brown) along with the Association of University Technology Managers (AUTM) announced a plan to facilitate access to university innovations with a clause ensuring global access to low-cost products by manufacturers for infectious diseases [30],[31],[32]. To date, the NIH, the Centers for Disease Control, and a private manufacturer (Najit Technologies) have also signed. Many publicly funded universities and research institutes, including the University of California, have not.

Working for the public good and with its resources, a university's most important contract with the public is the social one: to disseminate the knowledge and the innovation they yield. In times of economic difficulty, it becomes all the more critical that public funds are used maximally for the public good. In a 2006 report to Congress, the President's Emergency Plan for AIDS Relief (PEPFAR) reported that:

…in every case generics prices present an opportunity for cost savings; in some cases, the branded price per pack of a drug is up to 11 times the cost of the generic version…Every dollar that can be saved can be used to support additional prevention, care and treatment services. [33]

Future successes of international humanitarian programs, such as PEPFAR, may well hinge on current decisions being made regarding the patenting and licensing policies of publicly funded university research. The NIH has recently taken the lead in requiring that publications that follow from research sponsored by them be made publicly available by no later than 12 months after the date of publication to the National Library of Medicine's free-access digital archive, PubMed Central [34]; is there any reason why an analogous effort to broadly mandate Global Access Licensing should not now be pursued? To that end, like-minded scientists, students, and policy makers are urged to contact our campaign to see Global Access Licensing executed for all publicly funded research from California to Cameroon.

## Supporting Information

Alternative Language Summary S1 Spanish Translation of the Summary by CEC.
(0.03 MB DOC)
