# Supplementary material for: The Silent Epidemic of Exclusive University Licensing Policies on Compounds for Neglected Diseases and Beyond
Source: PLoS Negl Trop Dis. 2010 Mar 30;4(3):e570. doi: 10.1371/journal.pntd.0000570 (PMC2846921; doi:10.1371/journal.pntd.0000570)
Supplement: Alternative Language Summary S1 — Spanish Translation of the Summary by CEC. (0.03 MB DOC) [file pntd.0000570.s001.doc]

**Resúmen del autor**

Es común para universidades otorgar licencias exclusivas para sus desarrollos a una sola compañía manufacturadora. Esta práctica ha producido una epidemia silenciosa en la cual los precios de medicamentos son demasiados altos para pacientes pobres en países subdesarrollados. Muchas veces, estas medicinas fueron creadas inicialmente con fondos públicos. Una posible solución para este problema es una “Licencia Global de Acceso”, o GAL, la cual requeriría que los inventos de la universidad sean hechos disponibles a bajo costo a países de bajo y mediano ingreso. Por ejemplo, la Universidad de Colombia Británica (UCB) negoció recientemente una licencia en la que una nueva formulación de un antimicótico (Amphotericin B) se hará disponible a bajo costo para el tratamiento de leishmaniasis en países pobres. Utilizando la Universidad de California—el segundo patentado más numeroso del mundo—como un ejemplo, nosotros discutimos los problemas de licencias exclusivas otorgadas por universidades y el impacto potencial que podría tener la adopción generalizada de los principios de acceso global a través de todas universidades.

Author Summary done by Connie Chen.
